# Time-course relationship between environmental factors and microbial diversity in tobacco soil

**DOI:** 10.1038/s41598-019-55859-4

**Published:** 2019-12-27

**Authors:** Zhaobao Wang, Yan Yang, Yuzhen Xia, Tao Wu, Jie Zhu, Jianming Yang, Zhengfeng Li

**Affiliations:** 1 Energy-rich Compounds Production by Photosynthetic Carbon Fixation Research Center, College of Life Sciences, Qingdao Agricultural University, Qingdao, 266109 China; 2 Hongta Tobacco (Group) Co., Ltd., Yuxi, 653100 China; 3 China Tobacco Yunnan Industrial Co., Ltd, Kunming, 650231 China

**Keywords:** Biodiversity, Environmental microbiology

## Abstract

Soil physicochemical properties and microbial diversity both play equally important roles in tobacco cultivation. However, the relationship between these factors remains unclear. In this study, we investigated their correlations through the whole tobacco growth period, including the pretransplanting (YX-p), root extending (R), flourishing (F), and mature (M) stages in the Yuxi region of the Yunnan-Guizhou Plateau by measuring physicochemical properties and conducting 16S/18S rRNA analysis. The analysis demonstrated that the microbial community richness and diversity continuously changed along with the growth course of the tobacco. Multiple environmental factors showed a certain correlation with the diversity of microbial communities. Some bacteria could accumulate nitrogen during the growth stages, and the diversity of the bacterial community also increased when the content of organic matter rose. In addition, the water content and available K also influenced the diversity of the microbial community. The dynamic changes in soil physicochemical properties and enzyme activities gave rise to differences in the microbial community composition and structure, all of which affected the growth of tobacco. This study revealed the time-course relationship between environmental factors and microbial diversity in tobacco soil. An understanding of this relationship provides guidance for research on the interaction system of plants, soil and microbes and on improving plant yield and quality.

## Introduction

Tobacco is an important cash crop whose growth usually lasts 120 days. The growth course of tobacco can be divided into four major stages based on the growth period of the tobacco plants: the pretransplanting stage (P), the root extending stage (R), the flourishing stage (F), and the mature stage (M)^1^. The P stage refers to the period from sowing to transplanting seedlings to the field. The length of the P stage can differ because of different growth environments and seedling cultivation and growing techniques. The tobacco seedlings gradually acclimate for 5–7 days after they are transplanted into the field. Then, the stems, leaves and roots grow faster, and the requirements for fertilizer and water are significantly increased. This period is defined as the R stage. Next, tobacco plants enter the vigorous growth period called the flourishing stage (F). This stage is the most important stage and it greatly influences the yield and quality of tobacco. The M stage lasts until the harvest of tobacco^1–3^. The quality of tobacco is related to various factors, including climate, crop rotation pattern, soil properties, and soil microbes^4,5^; of these factors, the soil physicochemical properties play an important role in tobacco growth.

Generally, the contents of organic matter (Om) and total nitrogen (TN) in soil should be suitable in regions of tobacco cultivation. High concentration of TN induce the excessive growth of tobacco, which leads to low accumulation of secondary metabolites, including nicotine, phenols, terpenes, alcohols and lipids^6^. High TN does not facilitate the formation of optimal sugar-alkali and nitrogen-alkali ratios, resulting in an imbalance of chemicals in tobacco^7^. Moreover, during the continuous-cropping process, the formation of root exudates (RE) suppresses the diversity of soil microbes, inducing an imbalance in the microbial structure and affecting the soil enzyme activities^8^. The soil enzyme activities are mainly generated by microbes and are related to the decomposition of mineral elements in soil. For example, acid phosphatase (ACP) facilitates the release of phosphorus, while polyphenol oxidase promotes the formation of humus^9^. It is worth mentioning that the soil enzyme activities result from the integrative action of multiple microbes and other factors (including temperature, water content, nitrogen, phosphorus and crop root system), giving rise to their differences in soil enzyme activity in different regions or growth stages^6,10^.

In addition to soil physicochemical properties in tobacco growing, soil microbes also played a role in influencing various biogeochemical cycles on major nutrients and maintaining soil health^11^. The variety and quantity of microbes in tobacco-planting soil were abundant, and bacteria were predominant. As the active interface of the interactions among tobacco, soil and microbes, the microbial composition and structure of the tobacco root system are of great significance to the nutrient absorption and healthy growth of tobacco. The improvement of the action of soil microbes accelerates the decompositions of organic matter, increases the quantity of soil humus, facilitates the formation of soil granular structures, and enhances the release and accumulation of available nutrients^12–15^. Therefore, studying the succession pattern of the composition and structure of soil microbes provides important suggestions for promoting tobacco growth and improving tobacco quality^16,17^. However, most of the previous research on tobacco soil microbes has been conducted based on high-resolution DDGE electrophoretic strip analysis, and little data have been provided^18^, indicating the urgent need for further research on the microbial community structure of tobacco soil.

In recent years, research has mainly focused on single factors or one growth stage, including studies on studying soil fertility in different tobacco-growing areas in China^19–23^, such as the Provinces of Yunnan, Henan. Chen *et al*. investigated the changes of the main fertility indicators of five typical continuous cropping tobacco fields in Henan Province^24^. Bai *et al*. quantified the degree of spatial variability of soil properties in central China, and discussed the spatial structures exist in the soil properties at the scales in the study area^25,26^. Xu *et al*. reported the nutrient status of the tobacco soil of Yunnan Province before transplanting tobacco seedlings during 2002 and 2006^27^. Most of the previous studies were focused on describing soil fertility and characteristics, lacking systematic research and investigation on the relationship between the properties of tobacco soils and soil microbes. Some researchers have investigated the succession pattern of soil microbial community structure. For instance, Niu *et al*.^28^ researched how soil microbial communities shifted during tobacco cultivation under different rotation systems, concluding that both soil microbial communities during the fallow stage and tobacco selection shaped the communities of tobacco at the mature stage. Wang *et al*.^29^ explored the bacterial community structure and functional potential of rhizosphere soils as influenced by nitrogen addition and bacterial wilt disease under continuous sesame cropping, proposing that they had a significant impact on the structure of bacterial communities in rhizosphere soil and that signal transduction and translation could play an important role in preserving plant health. Chen *et al*.^30^ studied the mechanism by which organic fertilizer and effective microbes could mitigate the yield constraints of peanut continuous cropping in a red soil of southern China. However, these studies failed to study the relationship between the soil physicochemical properties and the microbial diversity of rhizosphere soils during the different growth stages of tobacco cultivation. Therefore, this study will focus on revealing the time-course relationship between environmental factors and microbial diversity in tobacco soil in the Yuxi region of the Yunnan-Guizhou Plateau. In this research, we analyzed the soil properties and succession pattern of soil microbe structures along the growth stages using 16S rRNA^31^ and 18S rRNA analysis^32^. Based on the results, a correlation between microbial diversity and soil physicochemical properties was proposed that could benefit soil improvement and fertilization strategy optimization during tobacco cultivation.

## Results

### Soil physicochemical characteristics

**Figure 1:**
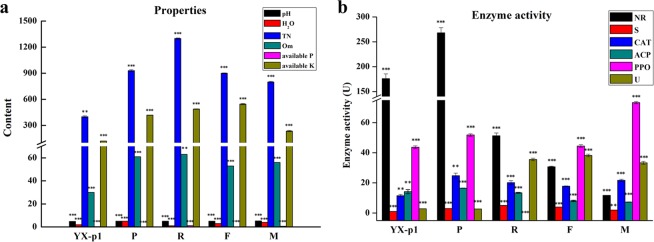
Physicochemical properties of soil samples. (**a**) pH; H_2_O, water content (%); TN, total N (mg/Kg); Om, organic matter (mg/Kg); available P (mg/Kg); available K (mg/Kg); (**b**) NR, nitrate reductase; S, sucrase; CAT, catalase; ACP, acid phosphatase; PPO, polyphenol oxidase; U, urease; (U), international unit of enzyme activity. One-sample t test was conducted, in which 95% of confidence interval was set. ^*, **, ***^ represent P value ≤ 0.05, 0.01, 0.001, respectively. All experiments were performed in triplicate.

As shown in Fig. 1, the pretransplant soil sample (named P) derived from the optimum plot (YX-2) had higher total N (TN), organic matter (Om), and available K (K)^33^ than those of the sample (named YX-p1) from the general plot (YX-1). Moreover, the enzyme activities of nitrate reductase (NR), sucrase (S), catalase (CAT), acid phosphatase (ACP), polyphenol oxidase (PPO), and urease (U) of P were all higher than those of YX-p1^34^. In this study, based on the superior physicochemical characteristics and microbial community richness/diversity (discussed below) of the optimum plot (YX-2) compared to those of the general plot (YX-1), a YX-2 plot was chosen for analysis of the time-course relationship between the soil physicochemical properties in tobacco cultivation and the soil microbial community structure. The soil physicochemical properties at the root extending (R), flourishing (F), and mature (M) stages in the optimum plot (YX-2) were detected. Among them, the soil pH^35,36^, water content (H_2_O), and enzyme activity of PPO increased along with the growth course of tobacco, while TN and the enzyme activities of NR, S, and ACP decreased along with the growth course^37,38^. In the mature stage (M), the water content and available P increased in contrast to those of the pretransplant soil sample (P)^39–42^. Conversely, the available K and enzyme activities of NR and ACP of M were significantly decreased compared to those of P. The other soil factors showed no significant change between M and P.

### Overview of microbial community diversity

For each sample, approximately 50,000 high-quality 16S rRNA^31^ gene sequences were obtained after high-throughput sequencing^43^. The rarefaction curve became flat, indicating that the amounts of operational taxonomic units (OTUs) reached saturation for all samples (Supplementary Fig. S1)^44,45^. All the coverages of the samples were extremely close to 1.0, suggesting that the coverage of the data was near complete (Supplementary Fig. S1)^46^.

**Figure 2:**
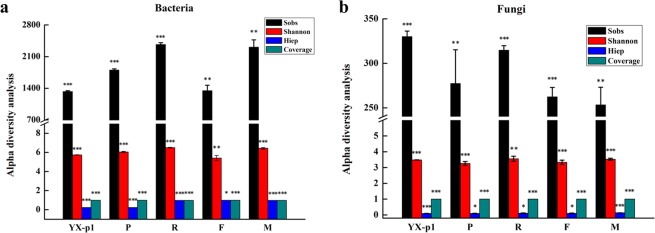
Alpha diversity analysis of the soil samples. Bacteria (**a**), fungi (**b**). One-sample t test was conducted, in which 95% of confidence interval was set. *, **, *** represent P value ≤ 0.05, 0.01, 0.001, respectively. All experiments were performed in triplicate.

The microbial community richness and diversity were analyzed using the alpha diversity analysis method^45^. In the pretansplant stage, the Sobs index of the optimum plot (P) were obviously higher than those of the general plot (YX-p1), indicating that the bacterial community richness was much higher in P^47^. The Shannon index of P was slightly higher than that of YX-p1. Meanwhile, the Heip index^46^ values of the two plots were similar to each other, suggesting that they had similar community evenness (Fig. 2a). In the optimum plot (YX-2), the three indices referring to community richness all showed an “increase-decrease-increase” variation trend along the root extending (R), flourishing (F), and mature (M) stages, respectively. The Shannon index showed the same variation trend along the growth time-course. As for the community evenness, the Heip index increased significantly after the pretransplant stage. However, it remained stable throughout all growing stages (Fig. 2a). The fungal community richness and diversity are shown in Fig. 2b, and they were found to change dynamically with the growth stages. The fungal community evenness remained almost identical in all the samples. All these data suggested that the microbial community richness and diversity continuously changed along the growth course of tobacco, which might be related to the soil factors at the different growth stages.

**Figure 3:**
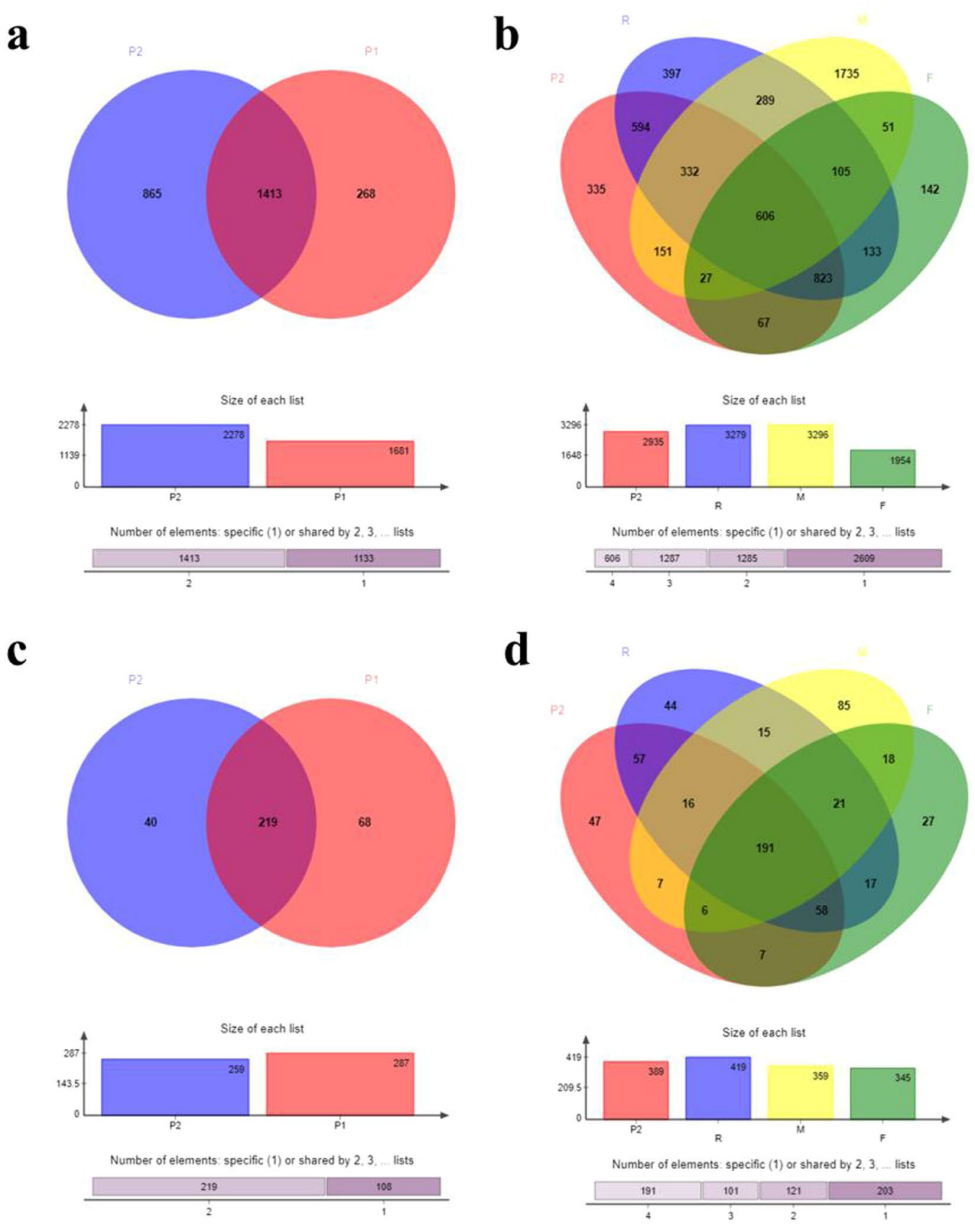
The differences of microbes composition between YX-p1 and P (**a** for bacteria, **c** for fungi), or P, R, F, and M (**b** for bacteria, d for fungi) using venn analyses. Venn diagram was used to count the number of common and unique species (such as OTU) in multiple groups or samples, and it could show the similarity and overlap of species in different samples intuitively.

As shown in Fig. 3, based on Venn analysis^48,49^, the number of unique OTUs in the optimum plot accounted for one-third to one-half of the total number of OTUs and was significantly higher than the proportion of special OTUs in the general plot. However, the special OTUs of the general plot were not found in the optimum plot, suggesting there were different microbial communities and structures in different soil regions. Therefore, it was deduced that the special OTUs or special microbial species gave rise to the differences in the microbial communities and structures^50^. Typically, the numbers of special bacterial species in R, F, and M were higher than that in P, demonstrating that when the proportion of special OTU species is higher, the diversity of the whole bacterial community of rhizosphere soil is increased, which gives rise to stronger resistance to the influence on tobacco roots on the bacterial community.

### Bacterial communities change with the growth stages

**Figure 4:**
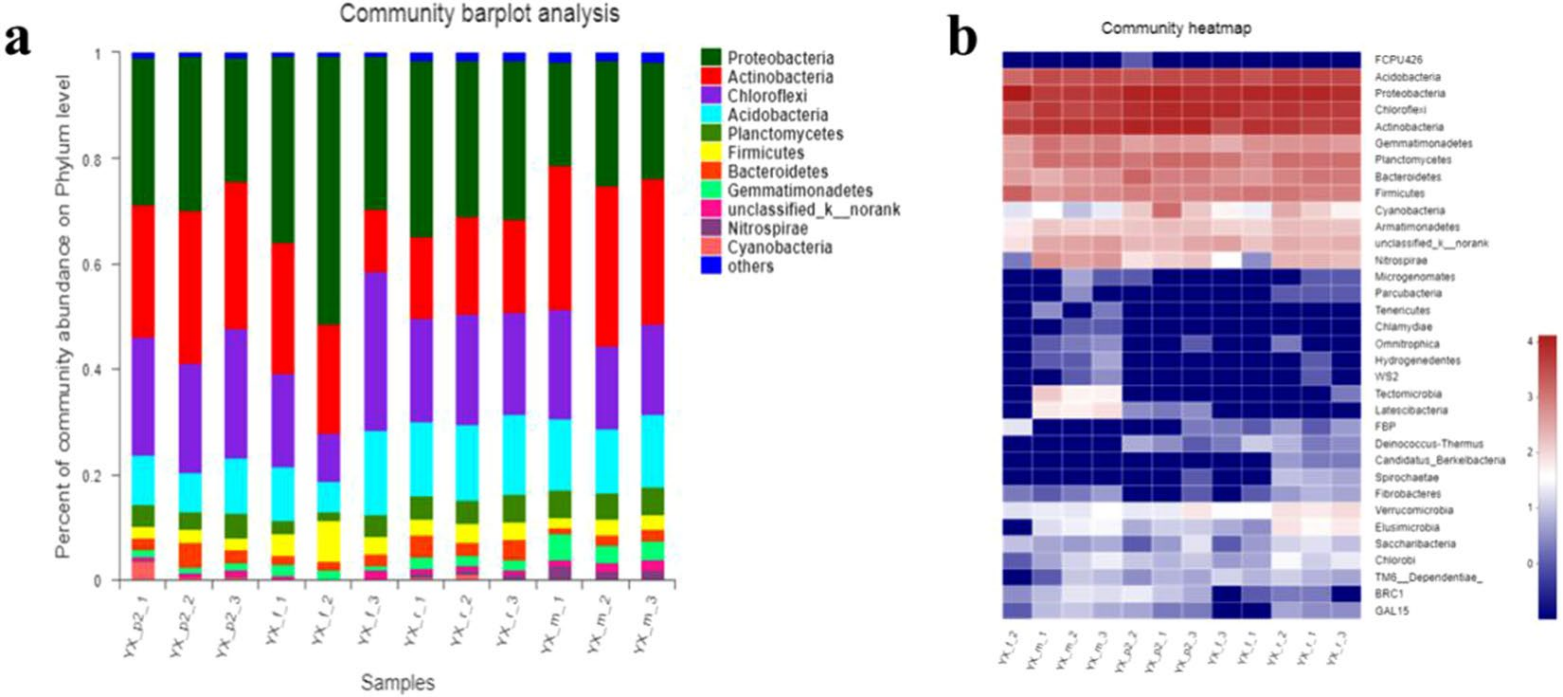
The bacteria community compositions and structures in different growth stages in YX-2 region. The bacteria community compositions at phylum level (**a**); the heatmap of bacteria communities (**b**). The community structure composition of different groups or samples on different taxonomic levels (such as domain, kingdom, phylum, class, order, family, genus, species, OTU, etc.) could be obtained based on taxonomic analysis. In this work, community Bar diagram and community heatmap were adopted respectively to show the characterizations of community composition of different samples. The microbial species and relative abundance contained in each sample at phylum taxonomic level were visually shown by the community Bar diagram. Clustering was carried out based on the similarity of species in different samples, and the results were presented on the community heatmap, allowing species with high and low richness to gather in blocks, and reflecting the similarity and difference of community composition of different samples using color changes.

Bacterial community composition and structure varied dynamically among the different growth stages (Fig. 4). At the phylum level, Proteobacteria, Actinobacteria, Chloroflexi, Acidobacteria, and Planctomycetes were dominant in all the soil samples but in different proportions. In P, Proteobacteria and Actinobacteria accounted for the majority of the bacteria. The great abundance of Acidobacteria in YX-p1 implied its oligotrophic characteristics; this kind of bacteria generally exists in an oligotrophic environment, which is in accordance with the low organic matter inYX-p1^51^. Planctomycetes, a kind of anaerobic ammonia-oxidizing archaea (AOA), are commonly found in anaerobic wastewater^52^. Therefore, the low proportion of Planctomycetes in the soil samples of this study indicates that most of the tobacco soils had a suitable oxygen supply^53^. At the genus level, the dominant bacteria included uncultured JG30-KF-AS9, Acinetobacter, Actinomycetes, Xanthomonas and Anaerolinea. The bacteria belonging to different families had obviously varying proportions in the samples of the YX-2 region as tobacco growth continued, among which Nocardiaceae and Anaerolinea accounted for a large proportion and Actinomycetes and DA111 accounted for a small proportion. In addition, it was found that all the dominant bacterial phyla included in the YX-1 region also showed high abundance, including Acidobacteria, Proteobacteria, Chloroflexi, Gemmatimonadaceae, Actinobacteria, Planctomycetes, Bacteroidestes, Firmicutes and Cyanobacteria. This indicated that all these abundant bacteria had great resistance to the effects of tobacco cultivation.

### Fungal communities change with the growth stages

**Figure 5:**
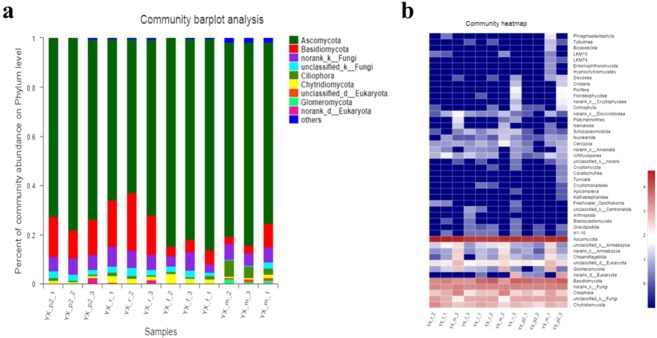
The fungal community compositions and structures in different growth stages in YX-2 region. The fungal community compositions at phylum level (**a**); the heatmap of fungal communities (**b**).

In the soil samples, smaller amount of fungi was identified, indicating that fungi also play a part in improving soil enzyme activity and maintaining the microbial community structure. Some of the fungi generate mycorrhiza with plant roots, which participate in root nutrient absorption^54,55^. The fungal categories that accounted for less than 0.01 percent were merged, and the dominant kinds of fungal phylum were obtained, including Ascomycota, Basidiomycota and Ciliophora (Fig. 5a)^56^. Cluster analysis was conducted on the heatmap of fungal communities with the top 20 abundances in different stages (Fig. 5b)^57^. It was found that a large number of the common microorganisms existing in the soil were Sordariales, Fusarium, Cladosporium, Boeremia, etc., most of which are Ascomycota fungi.

### Correlation analysis between soil physicochemical properties and microbial diversity

**Figure 6:**
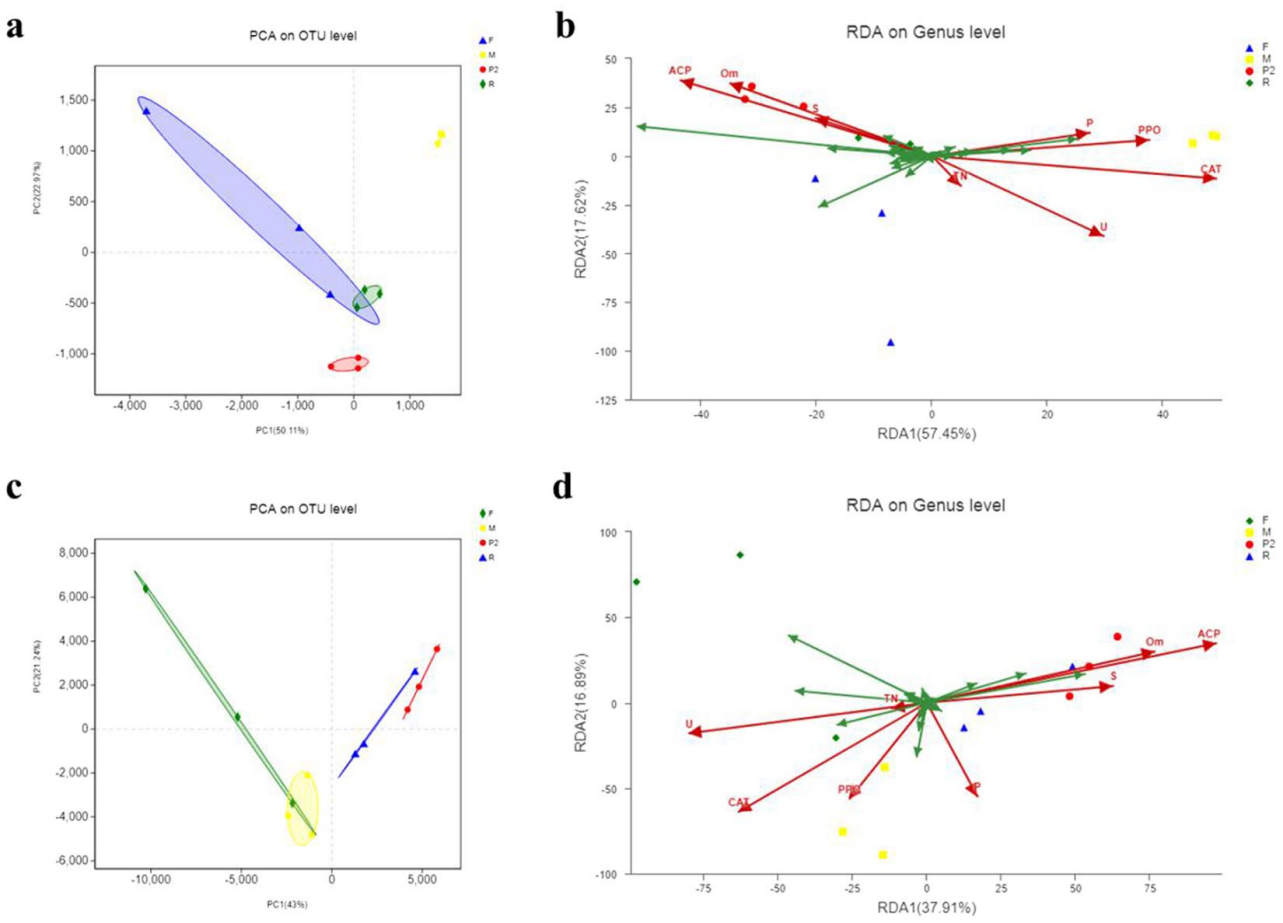
β diversity analysis of the microbial community of tobacco growth soils. PCA analysis of bacterial and fungi community, respectively (**a**,**c**); RDA analysis between bacterial or fungal community and environmental factors, respectively (**b**,**d**). β diversity, representing the comparison of microbial community composition, was usually adopted to evaluate the differences between different microbial communities. PCA (Principal Component Analysis), a technology adopted to simplify data analysis, was used to effectively figure out the most major elements and structures in the data. RDA (Redundancy Analysis), a kind of PCA analysis constrained by environmental factors, could reflect samples and environmental factors in the same two-dimensional ranking diagram, from which the relationship between sample distribution and environmental factors could be intuitively showed.

The bacterial community structures of YX-p1 and P showed great differences in the beta diversity analysis based on Bray-Curtis distance. Principal component analysis (PCA)^58,59^ was adopted to figure out the correlation between each of the samples in different groups, P2, F, R and M (Fig. 6a,c). Redundancy analysis (RDA)^60,61^ was conducted to reveal the relationship between the microbial community (P2, F, R and M) and the environmental factors (pH, H_2_O, TN, Om, available P, available K, NR, S, CAT, ACP, PPO, and U) (Fig. 6b,d). The diversity of the bacterial community increased when the concentration of organic matter rose. Meanwhile, the water content (H_2_O) and available K (K) also had a great influence on the diversity of the bacterial community, indicating that the appropriate water content would increase the nutrient content by activating the potassium-solubilizing bacteria^62^. In contrast, the soil physicochemical properties had less influence on the fungal community structure of YX-p1. However, available P, available K, water content, nitrate reductase (NR), acid phosphatase (ACP) and urease (U) had a greater influence on the fungal community structure of P than on that of the other treatments.

**Figure 7:**
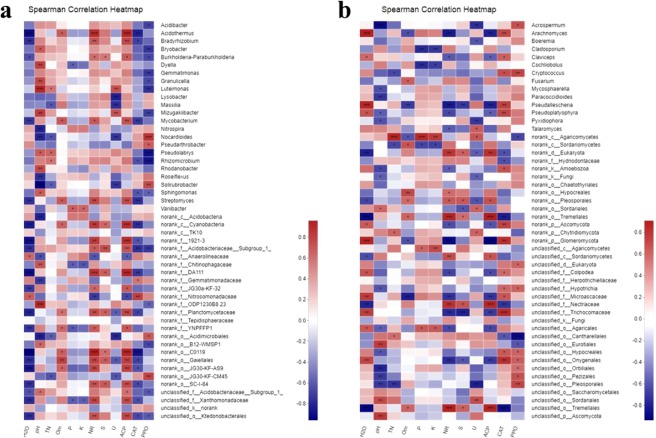
The correlations of environmental factors and bacterial (**a**) or fungal (**b**) communities of P using correlation Heatmap analysis. The correlation heatmap was used to show the relationship between microbial classification and environmental factors, by evaluating their correlation and representing the correlation coefficient between each microbe in the community and each environmental factor. Asterisks denote statistically significant t-test analysis. *, **, *** represent P value ≤ 0.05, 0.01, 0.001, respectively.

Correlation heatmap analysis^49,63,64^ was conducted to reveal the correlation between the structure of the microbial community and the environmental factors, which included nutrient elements and enzyme activity. This was aimed at providing suggestions for the inoculation of beneficial microbes to improve soil enzyme activity and promote the absorption of nutrients. Cluster analysis was conducted on the soil physicochemical properties with the 20 most abundant bacterial taxa. The correlations between the microbial community and the available K (K)/water content/urease (U) were consistent. The correlations between the microbial community and the environmental factors including NR, TN, available P and ACP showed consistent relevance^65^. Consistent relevance was also shown between the microbial community and the environmental factors including pH, organic matter (Om), catalase (CAT) and polyphenol oxidase (PPO) (Fig. 7a)^66,67^. In soil, K was correlated with the microbes of Nitrospira, Variibacter, Acidobacteria, and Anaerolineaceae, among which Acidobacteria can decrease the K content of soil^33^. High water content was shown to result in a reduction in the abundances of JG30-KF-CM45, Acidobacteria, Nitrospirasomonadaceae, Gemmatimonadaceae, TK-10, Variibacter, Pseudarthrobacter and Nocardioides, all of which play a role in maintaining the pH of soil, promoting the formation of organic matter, and maintaining the enzyme activity of CAT^33,34^. In summary, the presence of these microbes, including JG30-KF-AS9, Acidobacteriaceae-subgroup, DA111, Bryobacter, Bradyrhizobium, Planctomycetaceae, Gaiellales and Acidothermus, was detrimental to the enzyme activities in tobacco soil, which also resulted in a lack of available nutrients^39^. However, a high quantity of Gemmatimonadaceae, Anaerolineaceae, Nocardioides and Streptomyces was beneficial for maintaining high enzyme activities in the soil and decreasing the enzyme activity of urease^39,41–43^. The decrease in urease activity leads to the slow release of nitrogen fertilizer, with which available K and available P would be supplied to increase the available nutrient content in soil. These results would improve the growth of tobacco. Moreover, the relationships between the fungal community and environmental factors were irregular, indicating that fungi have an unclear effect on soil enzyme activity and the transformation of nutrients (Fig. 7b).

## Discussion

Soil environmental factors, including physicochemical properties and enzyme activities, play an important role in the tobacco growth process^34,35,50^. The tobacco soil containing 3% organic matter was considered to be high-quality soil and also had high catalase (CAT) activity^38,40^. In this work, it was determined that available potassium (available K) was mainly enriched by tobacco plants during the root extending stage (R) and the flourishing stage (F), respectively, based on the change in available K over the whole growth course. Total nitrogen (TN), organic matter (Om) and polyphenol oxidase (PPO) also had good consistency. In contrast, there was a negative correlation between acid phosphatase (ACP) and available phosphorus (available P)^68,69^. Regarding the dynamic changes in the enzyme activities, the activities of urease (U) and PPO changed little across the whole growth period of tobacco, while the activities of S and CAT decreased significantly; this decrease might be the reason for the failure of continuous cropping. Moreover, the increase in ACP activity was attributed to the continuing growth of tobacco.

The analysis of soil bacterial diversity revealed that the bacterial Shannon and Sobs indices of the pretransplant soil sample (P) derived from the optimum plot (YX-2) were higher than those of the pretransplant soil sample (named YX-p1) derived from the general plot (YX-1). The bacterial species found in P were not the same as the ones found in YX-p1. The number of special bacterial species in P was also higher than that in YX-p1. Moreover, it was deduced that different soil physicochemical properties could cause great differences in bacterial communities based on the analysis of the beta diversity. The microbial community structure formed by the bacteria including but not limited to the Acidobacteriaceae-subgroup, Bryobacter, Bradyrhizobium, Planctomycetaceae, Gaiellales and Acidothermus can reduce the activities of PPO and CAT^38,40,41^. However, maintaining a high quantity of Gemmatimonadaceae, Elev-16S-1332, Anaerolineaceae, Nocardioides, and Streptomyces in the bacterial community can benefit the activities of PPO and CAT^38,40,41^. Meanwhile, this bacterial community composition could reduce the activity of U, which is conducive to the slow release of nitrogen^37^; the addition of phosphorous and potassium with the nitrogen ensures the availability of nutrients for crop^34,35,40^.

There were few differences in the dominant fungal species between P and YX-1, including in the fungal abundance and variety. In this study, combined with a previous analysis, many of the same fungal species were found in different plots, indicating that fungi were not the main determinant distinguishing the suitability of the soil for planting. Based on the correlation analysis of fungal taxa and soil physicochemical properties, it was verified that PPO, CAT and TN could affect the fungal taxa, including Sordariales, Chaetothyriales, Hydnodontaceae, Tremellales, Pseudomycetes and Agaricales, present in the soil.

In conclusion, the study showed an obvious succession pattern of soil microbe structures through the tobacco growth stages, and this succession pattern corresponded to different soil physicochemical properties. The dynamic changes in soil physicochemical properties and enzyme activities gave rise to differences in microbial community composition and structure, all of which affected the growth of tobacco. The time-course relationship of the environmental factors in tobacco soil and the soil microbial diversity and the elucidation of how they together impact tobacco productivity provide guidance for research on the interaction systems of plants, soil and microbes and on improving plant yield and quality.

## Methods

### Soil sampling

Two sampling sites located in YuXi (YX), Yunnan Province (101°45′41“E, 24°5′28“N) were set for soil collection. According to the tobacco properties including nicotine, total sugar, total N, total K, total P contents, sugar-alkali ratio, nitrogen-alkali ratio, and potassium-chlorine ratio, *et al*. derived from the Cigarette Product Quality Testing Center of Yunnan China Tobacco Industrial Co., Ltd., the tobacco producing areas were divided into different quality grades. The two sites used in this study were annotated respectively as the general (YX-p1) and optimum (P) plots for tobacco cultivation in YuXi region, which were also set as the pretransplant stage of tobacco seedlings. For the optimum plot, rhizosphere soils were sampling at root extending, flourishing, and mature stages, respectively, which were named as R, F, and M (Supplementary Table S1). For each sample, five tobacco seedlings with the same growth status in the test field were selected randomly by sampling at five locations. For rhizosphere soil sampling, the roots were uprooted by shaking the roots, removing the loose soil at the roots, and collecting the soil at the roots with a sterile brush. To avoid the influence of additional fertilization, there were no fertilization practices at the sampling sites. Soil samples were collected in triplicate.

### Soil physicochemical properties

Samples were taken in triplicate each time, and the growth status of tobacco, root system and root with soil were photographed. The original soil samples were screened with 18-mesh screen to avoid fine roots, plant residues and stones from passing through the screen and stored in the refrigerator at −80 °C before subsequent experimental analysis was carried out. The soil texture was red loam. The basic physical and chemical properties and soil enzyme activities were determined after air drying, including pH, water content (H_2_O), total N (TN), organic matter (Om), rapidly available P, rapidly available K, sucrase (S), urease (U), catalase (CAT), polyphenol oxidase (PPO), nitrate reductase (NR), and acid phosphatase (ACP)^39–42^. The procedures of enzyme activities determination were listed in Supplementary File S2.

### DNA extraction

DNA was extracted from 0.5 g of soil samples (wet weight) using a Fast DNA Spin Kit for Soil (MP Biomedicals, USA)^43^. The final DNA concentration and purity were determined by NanoDrop 2000 UV-vis spectrophotometer (Thermo Scientific, Wilmington, USA), and genomic DNA quality was checked by 1% agarose gel electrophoresis. Primer sets 515F (5′-GTGCCAGCMGCCGCGG-3′) and 907R (5′-CCGTCAATTCMTTTRAGTTT-3′) were used for bacterial 16S rRNA gene amplification. Primer sets 817F (5′-TTAGCATGGAATAATRRAATAGGA-3′) and 1196R (5′-TCTGGACCTGGTGAGTTTCC-3′) were used for fungi 18S rRNA gene amplification. Barcode and adaptor sequences were ligated to the sequencing primers during the process of synthesizing, before the PCR was performed.

### PCR amplification

The PCR reactions were performed under the following conditions: initial denaturation at 95 °C for 2 min, 25 cycles of 95 °C for 30 seconds, 55 °C for 30 seconds, 72 °C for 30 seconds, and then a final extending at 72 °C for 5 min. The final annealing at 10 °C maintained for 5 minutes. Each sample was amplified in triplicate, pooled and purified using AxyPrepDNA Gel Extraction Kit (Axygen, USA). The concentration of purified PCR products was measured with QuantiFluor-ST system (Promega, USA).

### Data preprocessing and bioinformatics analysis

Purified amplicons were pooled in equimolar and paired-end sequenced (2 × 300) on an Illumina MiSeq platform (Illumina, USA). Barcode-tagged amplicons from different samples were mixed in equimolar concentration, and sent to the Majorbio Bio-Pharm Technology Co., Ltd. (Shanghai, China) for Miseq library construction and sequencing. The original fastq files were quality-filtered by Trimmomatic and merged by FLASH with the following criteria: (i) the reads were truncated at any site receiving an average quality score < 20 over a 50 bp sliding window. (ii) Sequences whose overlap being longer than 10 bp were merged according to their overlap with mismatch no more than 2 bp. (iii)Sequences of each sample were separated according to barcodes (exactly matching) and Primers (allowing 2 nucleotide mismatching), and reads containing ambiguous bases were removed. Operational taxonomic units (OTUs) were clustered with 97% similarity cutoff using UPARSE^70^ with a novel ‘greedy’ algorithm that performs chimera filtering and OTU clustering simultaneously. The taxonomy of each 16S rRNA gene sequence was analyzed by RDP Classifier algorithm against the Silva (SSU123) 16S rRNA database^31^ using confidence threshold of 0.7.

### Statistical analysis

Based on the results of OTU cluster analysis and taxonomic information, a series of in-depth statistical and visual analysis on community structure and phylogeny such as OTU generation, sampling adequacy analysis, abundance and diversity analysis, flora difference analysis, evolutionary tree analysis, etc. were carried out to screen soils with larger microbial abundance or more complex community structures.

For Illumina Miseq sequencing data, alpha diversity indices were calculated using the Quantitative Insights Into Microbial Ecology (QIIME)^47^. In the beta diversity analysis, the weighted UniFrac distance and Bray-Curtis distance were calculated using the “pure prime” packets by QIIME and ‘vegan’ package in ‘R’, respectively. Principal coordinates analysis was conducted to visualize the community similarity with the ‘vegan’ package in ‘R’. The alpha value of Kruskal-Wallis test was 0.05, and the threshold value of Logarithmic Linear Discriminant Analysis (LDA) score was 2.0. Using Welch’s t test with Bonferroni correction in ‘STAMP’, the differences of relative abundance among different treatments were analyzed. Principal component analysis was implemented by R programming language. The microbial community diversity indices, including number of bands, Shannon-Wiener index and Evenness index, were calculated as described before. Alpha diversity index of Illumina Miseq sequencing was tested by Welch’s t test, and the mean value between treatments was compared at a probability level of 0.05.

## Supplementary information

Supplementary Information
