# Supplementary material for: Time-course relationship between environmental factors and microbial diversity in tobacco soil
Source: Sci Rep. 2019 Dec 27;9:19969. doi: 10.1038/s41598-019-55859-4 (PMC6934738; doi:10.1038/s41598-019-55859-4)
Supplement: Supplementary file 1 — Supplementary Information [file 41598_2019_55859_MOESM1_ESM.pdf]

Time-course relationship between environmental factors and microbial diversity in tobacco soil

Zhaobao Wang[1], Yan Yang[1], Yuzhen Xia[3], Tao Wu[2], Jie Zhu[2], Jianming Yang[1*], Zhengfeng Li[2*]

[1]Energy-rich Compounds Production by Photosynthetic Carbon Fixation Research Center, College of Life Sciences, Qingdao Agricultural University, Qingdao, 266109, China.

[2]China Tobacco Yunnan Industrial Co., Ltd., Kunming, 650231, China

[3]Hongta Tobacco (Group) Co., Ltd., Yuxi, 653100, China

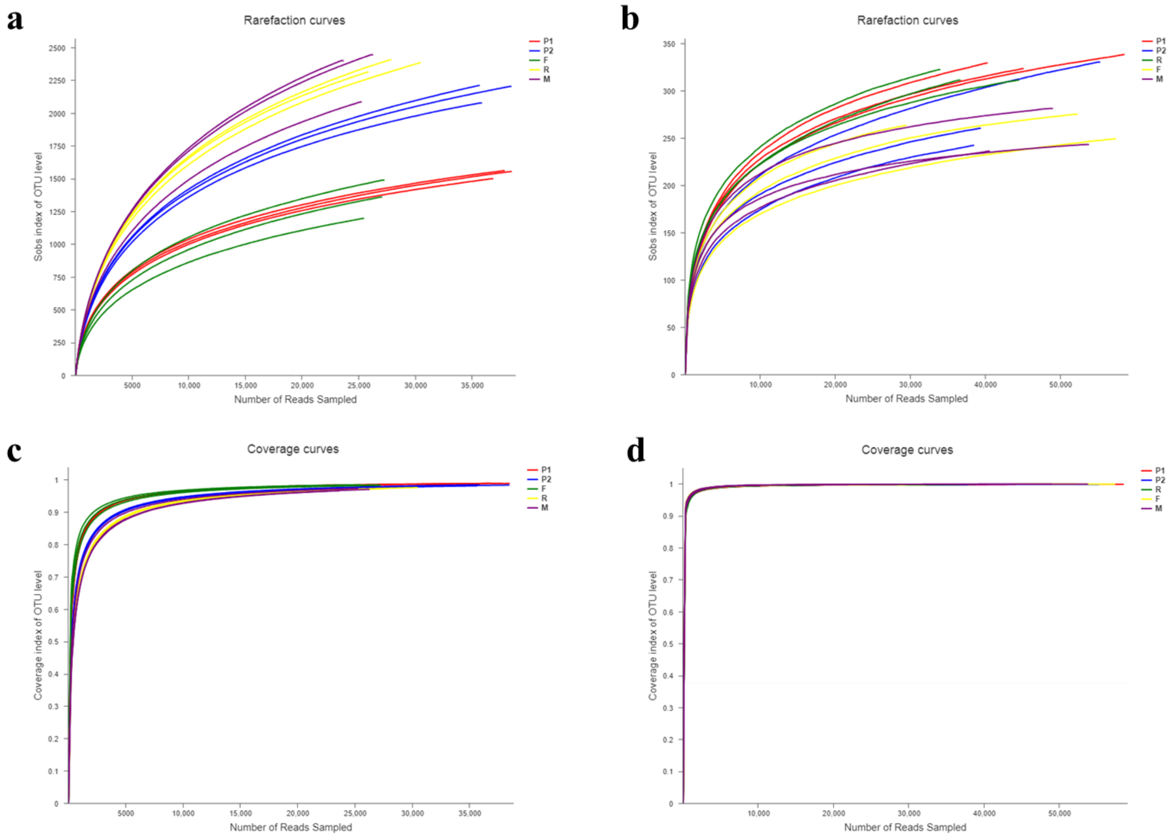

Figure S1. The rarefaction curves (**a** for bacteria, **b** for fungi) and coverage curves (**c** for bacteria, **d** for fungi) of the samples.

Table S1 The soil samples used in this study.

Location	Site name	Pretransplant stage	Root extending stage	Flourishing stage	Mature stage
Xinhua, Yuxi	YX-1*	YX-p1*	/	/	/
	YX-2*	P*	R*	F*	M*

* Two sites were selected in the previous work, named YX-1 and YX-2. The samples in the pretransplant stage of the two sites were named as YX-p1 and P, respectively. The samples of root extending, flourishing, and mature stages in the optimum site (YX-2) were named as R, F, and M, respectively.

File S2. The detailed procedures of enzyme activities determination.

Measure of soil enzyme activities

Determination of urease activity. A sample of 1 g soil producing 1 μg ammonium nitrogen in 24 h was set as 1 U. 0.25 ml toluene, 0.75 ml citrate buffer (pH, 6.7), and 1 ml of urea substrate solution were added to the 1 g soil sample and the samples were incubated. The formation of ammonium was determined spectrophotometrically at 578 nm and results were expressed as μg N g^{-1} dry soil. All determinations of urease activities were performed in triplicate, and all values reported were averages of the 3 determinations expressed on an oven-dried soil basis (105 ℃).

Determination of nitrate reductase activity. Nitrate reductase activity was calculated by the concentration of nitrite ions catalyzed by soil from nitrate. A sample of 1 g soil producing 1 μmol nitrite nitrogen in 24 h was set as 1 U. A sample of soil (5 g) was treated with 2 ml of absolute ethanol containing 2,4-dinitrophenol (DNP). The DNP concentrations desired was prepared by diluting an aliquot of the stock DNP solution (25 mg/ml) with ethanol. The alcohol was evaporated for 2 h, and the soil was treated with 10 ml of solution containing 500 μg NO_3^--N or 10 ml of 5 mM KNO_3. The sample and the contents were mixed by swirling for a few seconds to mix, stoppered, and held in the dark at 25 ℃ for 24 h. Then 40 ml 2.5 M KCl solution was added, following the sample was shaken for 30 min. Then the soil suspension was filtered. An aliquot (1 ml) of the resulting soil filtrate was transferred into a 50 ml volumetric flask and the NO_3^--N was determined by the calorimetric method of Griess-Illosvay.

Determination of sucrase activity. A sample of 1 g soil producing 1 mg glucose in 24 h was set as 1 U. A sample of soil added with 8% sucrose solution, phosphate buffer (PBS, pH 5.5) and methylbenzene was incubated at 37 ℃ for 24 h. Part of filtered supernatant was boiled for 5 min after 3,5-dinitrosalicylic acid was added. The formation of product was determined spectrophotometrically at 510 nm.

Determination of polyphenol oxidase activity. A sample of 1 g soil producing 1 μg purpurogallin in 1 h was set as 1 U. A sample of soil mixed with 1% pyrogallol solution was incubated at 30 ℃ for 2 h, after which citric acid-phosphate buffer (pH

4.5) was added. Then the mixture was extracted using ethyl acetate. The purpurogallin contained in the extraction was determined spectrophotometrically at 430 nm.

Determination of catalase activity. A sample of 1 g soil decomposing 1 g hydrogen peroxide in 1 h was set as 1 U. A sample of soil added with distilled water and 0.3% H_2O_2 was incubated in dark for 2 h, after which citric acid-phosphate buffer (pH 4.5) was added. Then saturated $KAl(SO_4)_2$ was added into the mixture, following it was filtrated into 1.5 M H_2SO_4 solution. The concentration of final product contained in the filtrate was determined spectrophotometrically at 240 nm.

Determination of acid phosphatase activity. A sample of 1 g soil producing 1 μmol phosphorus in 24 h was set as 1 U. A sample of soil was incubated for 1 h at 37 ℃ with 4 ml of modified universal buffer (MUB) (pH 6.5) and 1 ml of 0.115 M *p*-nitrophenylphosphate solution. After incubation, 1 ml of 0.5 M $CaCl_2$ and 4 ml of 0.5 M NaOH were added to the sample, which was followed by filtration. The concentration of final product was determined spectrophotometrically at 400 nm.
